# Non-invasive stimulation of the social brain: the methodological challenges

**DOI:** 10.1093/scan/nsaa102

**Published:** 2020-07-30

**Authors:** Tegan Penton, Caroline Catmur, Michael J Banissy, Geoffrey Bird, Vincent Walsh

**Affiliations:** Department of Psychology, Goldsmiths, University of London, London SE14 6NW, UK; MRC Social, Genetic and Developmental Psychiatry Centre, Institute of Psychiatry, Psychology and Neuroscience, King’s College London, London SE5 8AF, UK; Department of Psychology, Institute of Psychiatry, Psychology and Neuroscience, King’s College London, London SE5 8AF, UK; Department of Experimental Psychology, University of Oxford, Oxford OX1 3PH, UK; Institute of Cognitive Neuroscience, University College London, London WC1N 3AR, UK

**Keywords:** non-invasive brain stimulation, social perception, social cognition, state-dependent TMS, Autism Spectrum Disorder

## Abstract

Use of non-invasive brain stimulation methods (NIBS) has become a common approach to study social processing in addition to behavioural, imaging and lesion studies. However, research using NIBS to investigate social processing faces challenges. Overcoming these is important to allow valid and reliable interpretation of findings in neurotypical cohorts, but also to allow us to tailor NIBS protocols to atypical groups with social difficulties. In this review, we consider the utility of brain stimulation as a technique to study and modulate social processing. We also discuss challenges that face researchers using NIBS to study social processing in neurotypical adults with a view to highlighting potential solutions. Finally, we discuss additional challenges that face researchers using NIBS to study and modulate social processing in atypical groups. These are important to consider given that NIBS protocols are rarely tailored to atypical groups before use. Instead, many rely on protocols designed for neurotypical adults despite differences in brain function that are likely to impact response to NIBS.

## Introduction

Non-invasive brain stimulation (NIBS) refers to a range of techniques, including transcranial magnetic stimulation (TMS), transcranial electric stimulation (tES) and focussed ultrasound stimulation (tFUS), used to modulate brain excitability. Use of NIBS has increased significantly in recent years. This has enhanced our understanding of cognitive and perceptual processes (Miniussi *et al.*, 2012; Miniussi and Ruzzoli, 2013; Parkin *et al.*, 2015; Taylor, 2018) and enabled a new stream of intervention research (Rossi *et al.*, 2009; Miniussi and Vallar, 2011; Miniussi *et al.*, 2012; Perera *et al.*, 2016). Whilst of clear utility, this increasing experimental and applied research focus has been accompanied by questions regarding study design and generalisability of findings (Parkin *et al.*, 2015). In response, the field of brain stimulation has made efforts to strengthen experimental design. For example, several recent articles provide guidance on how to conduct well-controlled brain stimulation experiments (transcranial direct current stimulation [tDCS]—Ferrucci *et al.*, 2015; Woods *et al.*, 2016; TMS—Sandrini *et al.*, 2011; TMS-electroencephalography—Ilmoniemi and Kičić, 2010; Miniussi and Thut, 2010). In addition, there is increasing interest in understanding null results in NIBS studies and the mechanisms underlying NIBS effects (de Graaf and Sack, 2018; Thut *et al.*, 2018). One area of research that has benefitted from the use of brain stimulation techniques is social processing. Here, we review examples of the application of NIBS in this area of research and outline several key contributions of NIBS research to our understanding of social processing and its neural correlates; specifically, face processing, mirror responses and self–other processing. Whilst this review is not exhaustive, it highlights the utility of NIBS methods to study social processing.

Addressing more nuanced challenges facing social processing research using NIBS methods is important to allow for reliable interpretation of findings in neurotypical cohorts. It also allows us to tailor NIBS protocols to atypical groups with social difficulties. Therefore, we highlight several methods and techniques that may help to support the use of NIBS in both typical and atypical groups. Note, we assume that the reader has a working knowledge of commonly used NIBS techniques, but there are several useful reviews for a more detailed introduction (Walsh and Cowey, 2000; Walsh and Pascual-Leone, 2003; Wassermann *et al.*, 2008; Parkin *et al.*, 2015; Reed and Kadosh, 2018).

## How have NIBS studies contributed to understanding of social processing?

### Facial identity processing

One domain where NIBS has been used to explore social perception is the study of facial identity processing. Here, work has utilised both TMS and tES to explore this ability (e.g. Lafontaine *et al.*, 2013; Renzi *et al.*, 2013; Romanska *et al.*, 2015; Barbieri *et al.*, 2016). We specifically highlight the work elucidating the role of the occipital face area (OFA) in facial identity processing as a clear example of how using TMS can extend and support previous findings in facial identity research. Whilst beyond the scope of the current review, we also acknowledge the extensive body of work using NIBS to investigate processing of facial expressions (see Atkinson and Adolphs, 2011; Pitcher, 2019 for reviews in this area).

**Fig. 1:**
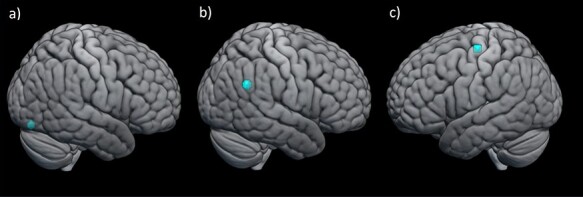
Commonly targeted stimulation sites in studies investigating face processing, self–other control and mirror responses. (a) right occipital face area; coordinates taken from Pitcher *et al.* (2007), (b) right temporoparietal junction; coordinates taken from Young *et al.* (2010), (c) left primary motor hand area; coordinates taken from Maegherman *et al.* (2019).

Influential models of face processing suggest the OFA contributes to early visual processing of faces (Haxby *et al.*, 2000; Calder and Young, 2005), with further processing relying on a distributed network of brain regions (Rossion, 2014). This model is supported by a combination of functional magnetic resonance imaging (fMRI), lesion and animal work (Atkinson and Adolphs, 2011; Rossion, 2014), but has been extended and tested through the use of NIBS methods (Atkinson and Adolphs, 2011; Pitcher *et al.*, 2011; Pitcher, 2019). Work by Pitcher *et al.* (2007) demonstrated the importance of the right occipital face area (rOFA) in processing facial features (Figure 1). Disruption of face discrimination abilities was observed after stimulation to the rOFA when facial features were varied, but not when the spacing between features was varied, suggesting a role for the rOFA in featural but not holistic face processing (Pitcher *et al.*, 2009; Solomon-Harris *et al.*, 2013). Furthermore, using double-pulse TMS (two single pulses of TMS applied close together in time), the authors demonstrated the time course of rOFA involvement. Specifically, rOFA TMS reduced face discrimination accuracy only when delivered 60 and 100 ms after stimulus onset. Ambrus *et al.* (2017a) extended these findings using TMS to explore the role of the rOFA in recognising different images of the same identity (Ambrus *et al.*, 2017b).

Collectively, these findings validate and extend models of face processing implicating the OFA in early face processing (Haxby *et al.*, 2000; Calder and Young, 2005). The work builds on fMRI studies by demonstrating a causal relationship between OFA activity and face processing (Rossion, 2014). It also supports findings from lesion studies that disruption to the OFA can impair face processing, whilst overcoming limitations of such studies (such as non-localised lesions making it difficult to infer site-specific effects, or cortical reorganisation following trauma limiting generalisability to a healthy brain). This work also builds on fMRI and lesion studies by demonstrating the time course of OFA involvement in face processing. Finally, the work demonstrates task, site and temporal specificity of brain stimulation effects. It is clear, therefore, that the use of NIBS has provided an important contribution to our understanding of the role of the OFA in face processing.

### Mirror responses

In the action domain, mirror neurons fire both when performing an action and when observing another agent performing the same, or a similar, action (Gallese *et al.*, 1996). It has been suggested that this ability to map observed movements onto the observer’s own motor representations may assists in understanding another’s actions (Rizzolatti and Sinigaglia, 2010), although a re-analysis of available data suggests mirror neurons instead respond to socially contingent actions (e.g. imitation; Cook *et al.*, 2014), with a potential role in action perception (Thompson *et al.*, 2019).

Research into mirror responses provides another example where NIBS studies have complemented animal, imaging and lesion studies to further understanding of the neural basis of social processing (Keysers *et al.*, 2018). Research in non-human primates identified mirror neurons in area F5 (homologue of ventral premotor cortex in humans) and inferior parietal regions (Casile, 2013). In humans, fMRI revealed increased activation in these regions during action observation and execution (Caspers *et al.*, 2010). In NIBS studies, mirror responses are indexed by measuring muscle responses to single-pulse TMS delivered over the primary motor cortex (motor evoked potentials [MEPs]; Figure 1). Changes in MEP amplitudes are thought to index motor cortex excitability, with larger amplitudes indicative of greater excitability (Fadiga *et al.*, 1995). Strafella and Paus (2000) demonstrated a muscle-specific increase in excitability to observation of different actions, coupled with a muscle-specific reduction in cortical inhibition and facilitation (indexed by reduced response to short intracortical inhibition and intracortical facilitation, respectively). By demonstrating the muscle-specific nature of mirror responses, these findings go beyond what had previously been demonstrated using fMRI. Subsequently, extensive NIBS work perturbing different brain regions has demonstrated the anatomical specificity and functional role of brain regions involved in producing mirror responses (Keysers *et al.*, 2018).

NIBS studies have also shed light on connectivity patterns between regions involved in mirror responses and their likely origin. For example, Catmur *et al.* (2011) showed that connectivity between mirror response regions can be altered through associative learning. Initially, a conditioning pulse applied to either the dorsal or ventral premotor cortex facilitated MEP responses from M1 representations of index and little finger muscles after observation of index or little finger actions, respectively. After counter-mirror training to alter learned associations between observed and executed actions (where participants move their index finger in response to observed little finger movements and vice versa; Catmur *et al.*, 2007), mirror responses were significantly reduced. This reduction was amplified following conditioning pulses to the premotor cortex, supporting the idea that the mirror system can adapt through associative learning (Cook *et al.*, 2014) and demonstrating the role of premotor–M1 connections in such associations.

Collectively, these NIBS studies demonstrate the causal role of a group of brain regions, and connectivity between these regions, in mirror responses and lend support to key theories such as associative learning accounts of mirror response origin. These studies also demonstrate muscle-specific responses to action observation, and hence mirror responses, more directly than is possible using neuroimaging.

### Self–other processing

During social interaction it can be important to enhance representation of another person and suppress representation of the self (e.g. in order to represent another’s beliefs when they differ from your own). Conversely, it can also be beneficial to suppress representation of another and enhance representation of the self (e.g. to inhibit imitation of another). This ability to selectively modulate representations of the self and the other is known as self–other control and is thought to play a key role in several social processes including empathy, perspective taking and theory of mind (Ward and Banissy, 2015; de Guzman *et al.*, 2016). The medial prefrontal cortex (mPFC) and the temporoparietal junction (TPJ) have been linked to this process through a body of fMRI work (e.g. Brass *et al.*, 2009). The use of NIBS has allowed the causal link between the TPJ and self–other control to be established. For example, Costa *et al.* (2008) and Young *et al.* (2010) both showed that 1 Hz repetitive TMS (rTMS) to the right TPJ (rTPJ; Figure 1) disrupts performance on theory of mind task. Similarly, Wang *et al.* (2016) showed that double-pulse TMS to the right posterior TPJ also disrupted performance on a perspective-taking task. Furthermore, rTMS delivered at a theta frequency (6 Hz) relative to alpha (10 Hz) facilitated embodied perspective taking, highlighting the role of theta oscillations in this process (Gooding-Williams *et al.*, 2017).

Studies have also employed tDCS to investigate the role of the TPJ in self–other control. For example, Santiesteban *et al.* (2012) demonstrated that anodal tDCS to the rTPJ selectively improved performance on tasks requiring self–other control (imitation–inhibition and perspective taking) relative to a task requiring self-referential processing. No differences in task performance were found between cathodal stimulation and sham. This effect of improved self–other control following anodal tDCS to the rTPJ was subsequently replicated by Santiesteban *et al.* (2015), who also showed a similar pattern of results for left TPJ stimulation (Hogeveen *et al.*, 2014). Collectively, these findings highlight the role of the TPJ in self–other control. In addition, they demonstrate that modulation of social processing can be achieved, and replicated, using tDCS methods (see Sellaro *et al.*, 2016 for review on tDCS in social processing research).

Whilst NIBS research has clearly enhanced understanding of the role of the TPJ, further research is needed to understand the role of the mPFC. It is commonly thought that ventral regions of the mPFC are involved in self-referential processing, whereas dorsal regions are involved in representing others (see van der Meer *et al.*, 2010; Denny *et al.*, 2012 for meta-analyses). However, Nicolle *et al.* (2012) suggested that the mPFC is organised with respect to task-relevance, thus challenging prevailing accounts of mPFC organisation (also see Cook, 2014). They argued that ventral regions of the mPFC keep track of task-relevant information (e.g. information about the self during a self-relevant trial), whereas more dorsal regions of the mPFC keep track of task-irrelevant information (e.g. information about the self during an other-relevant trial). Use of more focal NIBS techniques (such as TMS) is one way to test contrasting accounts of brain function in social processing. However, we are generally limited to stimulating areas near the cortical surface. Targeting deeper regions often requires higher intensity stimulation, which impacts focality of the electric field. Thus, in order to test accounts regarding the role of deeper or less accessible brain structures in social processing (e.g. mPFC), we must first overcome several challenges associated with using NIBS in social processing research.

## Challenges using NIBS to study social processing

Whilst the above examples highlight successes of using NIBS to modulate social processing, there are also a number of challenges. The remainder of this paper will discuss key challenges facing researchers using NIBS to study social processing in neurotypical and atypical populations. This section is not an exhaustive list of limitations, but rather highlights several challenges that are particularly problematic.

### Depth of regions of interest

With most brain stimulation methods, we are only able to target shallow cortical regions (Kammer, 1998; Roth *et al.*, 2007). This can be problematic for many areas of study, but is particularly challenging when investigating social processing that relies on networks encompassing subcortical regions. For example, processing of facial emotions requires a distributed network including cortical regions such as the ventromedial prefrontal cortex and somatosensory cortex, less accessible structures such as the fusiform gyrus, and subcortical regions such as the amygdala and insula (Adolphs, 2002; Fairhall and Ishai, 2006). If we could reliably target deeper regions, we may be able to further understand the role of, and connectivity between, different regions within networks responsible for social processing. With TMS, it is possible to stimulate subcortically using alternative coil types to the commonly used figure-of-eight coil. However, the increased current spread makes approaches like this unsuitable for most studies as it reduces the focality of stimulation. Unintended cortical surface stimulation is also a problem with such techniques. Collectively, these issues make it difficult to make inferences regarding the function of more specific, deeper brain regions (for comparison of induced electric field, see Deng *et al.*, 2013; Lu and Ueno, 2017). Therefore, methods that allow focal stimulation of deeper regions would be very useful in social processing research.

Currently, it may be possible to overcome this issue using an indirect stimulation protocol (see Wang *et al.*, 2014; Kim *et al.*, 2018 for examples of network stimulation effects in associative and episodic memory). Many studies have shown that the effects of TMS can alter activity in non-targeted areas of a network activated during a given task (see Ruff *et al.*, 2009 for review). This approach has been used to modulate interoceptive processing through direct stimulation of cortical regions implicated in the interoception network (dorsolateral prefrontal cortex) that results in indirect activation of subcortical regions in the network (anterior insula; Mai *et al.*, 2019). Similar network effects have been shown in face processing whereby stimulating the rOFA alters fusiform face area activity, and stimulating the posterior superior temporal sulcus (pSTS) alters amygdala activity (Pitcher *et al.*, 2014, 2017). Thus, it may be possible to exploit such effects to modulate activity in less accessible brain areas (i.e. targeting cortical sites to indirectly modulate less accessible regions). Whilst useful, this potential for indirect effects of NIBS can also make it difficult to interpret regional involvement in a given process (Coll *et al.*, 2017).

One thing that several of these studies have in common is the use of imaging methods to verify change in subcortical network activation. Use of imaging methods is important to ensure that indirect stimulation protocols are indeed modulating these less accessible regions. This may not always be the case when targeting cortical regions that are implicated in several networks. The flexible hub theory (Cole *et al.*, 2013) posits that brain areas are involved in multiple networks and that brain state will determine whether interaction with one network is privileged over another. Regions can flexibly interact with different brain networks depending on the nature of a participant’s task. Thus, if a brain region is part of more than one functional network (e.g. involved in both perception and memory networks), caution is required to ensure that tasks used capture the role of the region in the specific functional process of interest. In such cases, confirmation of network effects with neuroimaging would permit stronger inferences to be drawn.

In addition to indirect effects of NIBS, it may be possible to target deeper regions in the future using two emerging techniques. First, low-intensity tFUS is a form of NIBS relying on pressure produced by ultrasound waves to modulate brain activity (Tyler *et al.*, 2018; Darrow, 2019; di Biase *et al.*, 2019). Importantly, this technique is thought to be able to stimulate subcortically whilst preserving spatial focality. This is because the acoustic focus (where the acoustic energy is greatest) can be steered towards deep sites whilst keeping the size of the stimulated area as small as possible (Legon *et al.*, 2018; Folloni *et al.*, 2019). Accordingly, this also reduces the degree of unintended cortical stimulation (i.e. stimulation of superficial sites when targeting less accessible regions). Thus, tFUS provides a useful alternative to other deep NIBS methods (e.g. deep TMS using H- or double-cone coils), which suffer from a depth-focality trade off (Deng *et al.*, 2013; Lu and Ueno, 2017). Preliminary data in humans have shown that tFUS can alter unilateral thalamic activity (Legon *et al.*, 2018). In addition, tFUS over the primary somatosensory cortex modulates somatosensory evoked potentials and behavioural performance on a sensory discrimination task (Legon *et al.*, 2014), thus highlighting the potential of tFUS techniques to modulate behaviour in humans. However, tFUS is still in its infancy and more research into safety thresholds and mechanisms of action is needed prior to use in social processing research (Pasquinelli *et al.*, 2019). Once better understood, tFUS may provide a useful tool to modulate deeper regions in social brain networks.

Transcranial temporal interference stimulation (tTIS; Grossman *et al.*, 2017) may also overcome unintended cortical stimulation whilst being able to target less accessible regions. This method applies two different high-frequency electrical fields to the brain via surface electrodes. Applying current at such high frequencies (in the kHz range) is not thought to modulate neural oscillations (Hutcheon and Yarom, 2000). However, at the point where the frequencies overlap, an amplitude-modulated field is created. This waveform oscillates at a slower frequency, the rate of which is equal to the difference between the frequencies generated by the two surface electrode pairs. Depending on surface electrode placement, it may be possible for this overlap to occur in deeper brain regions, thus modulating activity of deeper areas. Importantly, because the waveforms are not overlapping on the cortical surface, activity of more superficial areas is unaffected. This method may therefore be useful for modulating deeper areas of social brain networks. tTIS has been shown to modulate focal cortical and subcortical regions in rats (Grossman *et al.*, 2017), and feasibility of this technique in humans has recently been addressed using computational modelling approaches (Grossman *et al.*, 2018; Rampersad *et al.*, 2019). However, more work is needed to understand the mechanisms of action, feasibility and safety of this approach in humans.

### Overlapping and neighbouring brain regions

When using NIBS, it can be difficult to dissociate the role of a region of interest in task performance from the role of other neighbouring regions. This is due to both network activation and current spread to other neighbouring regions. For example, different regions of the TPJ are involved in different cortical networks. The anterior TPJ shows connectivity with the ventral attention network (Corbetta and Shulman, 2002) and is implicated in both social and non-social processing, whereas the posterior TPJ shows connectivity with the social cognition network and is primarily implicated in social processing (Mars *et al.*, 2011; see Krall *et al.*, 2015 for meta-analysis). Whilst associated with different processes, these regions are topographically close. Thus, targeting just one with NIBS techniques becomes challenging. As such, it is important to ensure that when investigating the effects of brain stimulation on regions involved in social processing, we do not use tasks that also rely on alternative networks that include anatomically close regions. Conversely, it is also possible to use control tasks that may differentially activate these alternative networks. For example, Santiesteban *et al.* (2017) demonstrated that domain-general attentional processes, rather than implicit mentalising, were modulated by rTMS to the rTPJ. By investigating both domain-general and domain-specific effects of rTPJ stimulation, the authors were able to shed light on rTPJ involvement in social processing. It can be difficult to design tasks that allow for this dissociation, but it is essential if we are to understand how modulation to an area affects social processing specifically, rather than more general processing.

It may also be possible to account for anatomical specificity of an effect by stimulating the region of interest and other anatomically close control regions. If task behaviour is modulated by stimulation to one site but not another nearby site, this would provide stronger evidence that modulation of the region of interest, rather than neighbouring regions, is driving the effect (subtractive inference; Walsh and Cowey, 2000). Coupling such protocols with imaging methods would further enhance our knowledge of anatomical specificity. It is also possible to record network activation following plasticity-inducing NIBS protocols (e.g. network activation recorded prior to and following a theta-burst TMS protocol). Whilst this does not overcome the issue of stimulating overlapping or neighbouring regions, it does allow for regional and network changes in activity to be detected.

**Fig. 2:**
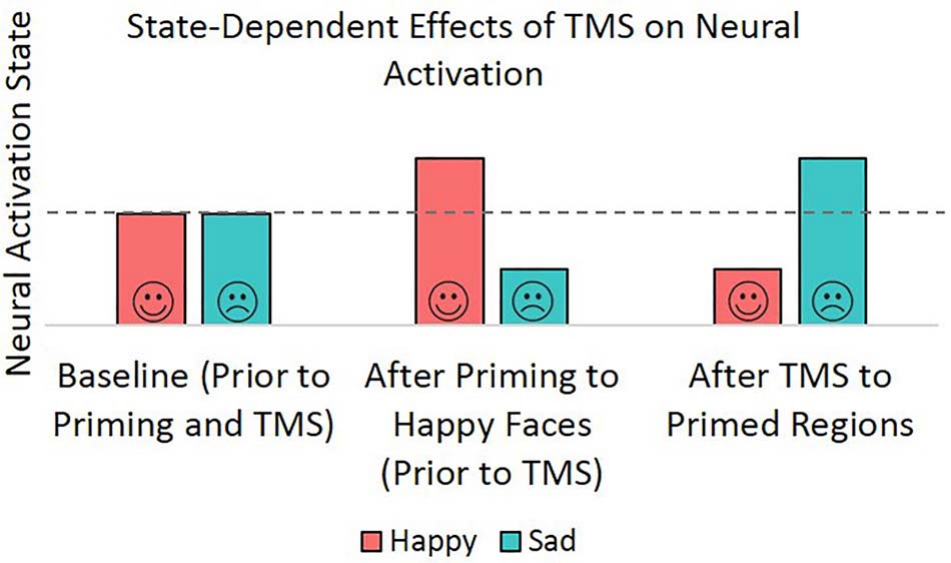
Theoretical approach to exploiting state-dependent effects of NIBS in social processing research. Left to right: neural activation of representations of different facial emotions is initially at a baseline level. Activation of neurons coding for a particular facial emotion is then manipulated through use of priming. Following subsequent TMS, activity of the primed neurons (i.e. those coding for happy faces) may be inhibited compared to baseline, whereas activity of unprimed neurons (coding for sad faces) may be facilitated. This theoretical pattern of results is in line with empirical evidence in the visual perception domain whereby TMS facilitates activity of less active neural populations (Silvanto *et al.*, 2008).

One way to potentially overcome this issue is to exploit state-dependent effects of NIBS. Brain stimulation effects are influenced by the state of the brain at the time of stimulation (Silvanto *et al.*, 2007). For example, in visual perception, researchers have been able to selectively influence the behavioural outcomes of brain stimulation by altering the brain state at the time of stimulation (e.g. Cattaneo and Silvanto, 2008; Silvanto *et al.*, 2008; Silvanto and Muggleton, 2008). Silvanto *et al.* (2008) showed that priming area V5 of the visual cortex (*vs* the vertex) with 1 Hz inhibitory rTMS resulted in facilitation of motion detection performance when receiving online TMS. In contrast, online TMS to area V5 disrupted motion detection performance when activity in this area was not suppressed (offline rTMS delivered to vertex control site). This study shows that it is possible to change the nature of the effects of stimulation by influencing the brain state at the time of stimulation (Cattaneo and Silvanto, 2008; Silvanto and Muggleton, 2008). Endogenous baseline activity has also been shown to partially explain variability in response to TMS (Pasley *et al.*, 2009; for theoretical framework see Silvanto and Cattaneo, 2017). Silvanto and Pascual-Leone (2008) described the potential utility of exploiting state-dependent effects of NIBS in perceptual studies to selectively target specific brain networks. It may be possible to apply a similar approach to social processing research. In theory, this approach may provide a way to selectively activate networks involved in social processing whilst limiting modulation of other contiguous networks that may otherwise be influenced by NIBS (Figure 2).

One example comes from Mazzoni *et al.* (2017) who exploited state-dependent effects of TMS to investigate areas involved in representing affective body kinematics (using point-light displays). Working on the premise that single-pulse TMS facilitates less active/excitable neural populations (Silvanto and Pascual-Leone, 2008), Mazzoni *et al.* (2017) used an adaptation paradigm where participants were exposed to happy or fearful adapters prior to a judgement task. During the judgement task, participants indicated whether a target display was happy, fearful or neutral. Participants were faster to respond to adapter-incongruent targets when receiving no TMS, TMS to an active control site or TMS to the pSTS. However, this effect was abolished for fearful displays only when receiving TMS to the anterior intraparietal sulcus (aIPS). This suggests that neural populations in the aIPS code affective (fearful) kinematic profiles and highlights the utility of state-dependent effects of TMS in social processing research (see also Cattaneo *et al.*, 2010, 2011; Jacquet and Avenanti, 2015 for state-dependent studies of action observation; and Ambrus *et al.*, 2017b, 2019 for state-dependent studies of face processing). Thus, state manipulations may provide a useful method to understand the role of regions/networks in social processing, and to overcome limitations associated with stimulation of overlapping/neighbouring regions.

## Use of NIBS in autism and other atypical groups

There is a growing body of work assessing the potential use of NIBS in clinical disorders (for reviews see Machado *et al.*, 2008; Kim *et al.*, 2009; Wassermann and Zimmermann, 2012; Schulz *et al.*, 2013). Several studies have also shown promising results using NIBS to modulate social processing in atypical groups (see Boggio *et al.*, 2015 for review). However, the research in this area is limited. It is also important to consider that, in addition to the key challenges mentioned in the previous section, there are several additional challenges facing NIBS studies of social processing in atypical groups. These are important to consider given that NIBS protocols are rarely tailored to atypical groups. Instead, research in atypical cohorts often relies on protocols shown to be effective in neurotypical groups. In the next section, we discuss challenges facing studies of social processing in atypical groups using NIBS. We will use the case of Autism Spectrum Disorder (hereafter ‘autism’) as an example throughout.

Autism is a neurodevelopmental disorder characterised by social difficulties and rigid and repetitive behaviours (American Psychiatric Association, 2013). In addition to these core symptoms, people with autism often exhibit motor control difficulties (Gowen and Hamilton, 2013) and have significantly higher rates of neuropsychiatric disorders such as depression and anxiety (Hollocks *et al.*, 2019). Research investigating ways to ameliorate social difficulties associated with autism or co-occurring disorders and traits (e.g. social anxiety, alexithymia) is therefore an important area of study for researchers investigating social processing and for the autistic community (Pellicano *et al.*, 2014).

Atypical groups may also benefit greatly from social interventions in neurotypical participants. For example, many autistic individuals find social situations challenging due to difficulties interpreting social cues of others. However, social situations may also be challenging due to a failure of neurotypical controls to interpret social cues of their autistic peers (Brewer *et al.*, 2016; Edey *et al.*, 2016). Thus, interventions must account for both autistic and neurotypical difficulties in order to improve social interactions across these cohorts. NIBS techniques may provide a useful tool to understand and ameliorate social difficulties in both typical and atypical populations. However, use of such techniques in people with autism and other disorders should be approached with caution (Wassermann and Lisanby, 2001; Bersani *et al.*, 2013; Kuo *et al.*, 2014; Oberman *et al.*, 2015).

### Stimulation protocols in typical and atypical populations

#### Network recruitment and connectivity

Multiple papers highlight high variability in response to brain stimulation in neurotypical adults and the need to individualise or tailor protocols to achieve maximal gain in both typical and atypical groups (for review see Krause and Cohen Kadosh, 2014). However, in practice, many studies investigating social perception in atypical groups are reliant on findings from the neuroptyical literature to inform protocols. This is problematic as it assumes that what holds in a neurotypical population will directly apply to atypical populations (Walsh and Pascual-Leone, 2003). This is important when considering the use of NIBS in atypical groups such as those with autism. Hanson *et al.* (2013) found that autistic participants showed different connectivity patterns during social processing tasks relative to neurotypical controls. This is consistent with other findings suggesting general atypical connectivity in autistic cohorts (Rubenstein and Merzenich, 2003; Assaf *et al.*, 2010). Importantly, this difference was not uniform across tasks. Participants with autism showed similar connectivity patterns to neurotypical controls when face processing networks were recruited, but not when theory of mind or action understanding networks were recruited (Hanson *et al.*, 2013). Collectively, these findings highlight different network recruitment and connectivity patterns in participants with autism relative to neurotypical controls. NIBS studies investigating social processing in these groups should, therefore, take this into account when selecting target sites or when designing paradigms to investigate connectivity patterns in participants with autism. Importantly, we cannot assume that stimulation to target sites shown to modulate social processing in neurotypical adults will modulate social processing in the same way in atypical groups.

#### Neurotransmitters

Atypical inhibition in the brain has been proposed as a common candidate endophenotype for a range of disorders (Marín, 2012). In autism, atypical GABAergic activity in the brain is observed due to a multitude of factors including reduced γ-aminobutyric acid (GABA) synthesis and reduced number of GABAergic receptors (for reviews see Rubenstein and Merzenich, 2003; Blatt and Fatemi, 2011). Atypical inhibition in autism may also results from atypical N-methyl-D-aspartate (NMDA) receptor activity (Lee *et al.*, 2015). In line with the above, atypical plasticity profiles have been observed across a range of disorders including schizophrenia and autism (e.g. Bourgeron, 2015; Hall *et al.*, 2015; Forrest *et al.*, 2018). These findings are important given that several NIBS techniques are thought to work by influencing NMDA and GABAergic activity and increasing plasticity in targeted regions (Liebetanz *et al.*, 2002; Huang *et al.*, 2007; Stagg *et al.*, 2009; Bachtiar *et al.*, 2015). Therefore, modulating these systems in the atypical brain may not have the same outcome as in a neurotypical brain. Indeed, atypical plasticity following rTMS in participants with autism has been observed (Oberman *et al.*, 2010, 2012). Thus, whilst interventions targeting these neurotransmitters in atypical groups may be useful, it is important to first tailor such interventions to the intended cohort.

One way to achieve this is through testing physiological and behavioural responses to NIBS techniques in atypical cohorts. This can be done by borrowing protocols from studies addressing this in neurotypical controls (Walsh and Cowey, 2000; Jacobson *et al.*, 2012; Krause and Cohen Kadosh, 2014; Parkin *et al.*, 2015; Reed and Kadosh, 2018). Ideally, this should be done prior to attempts to induce long-term changes in atypical groups using NIBS. A good example of work in atypical groups comes from Hoy *et al.* (2014) who showed dose-dependent effects of tDCS on working memory in participants with schizophrenia. Such work is important to ensure the safety of participants undergoing interventions and to increase the likelihood that participation is worthwhile for these groups. NIBS interventions can span months and require regular lab visits. Regular visits may be draining for atypical groups for many reasons (e.g. unknown social situation, anxiety when using public transport, etc.). Therefore, the time and energy cost to the participant must be taken into account when engaging atypical groups in interventions. Understanding how NIBS affects these groups, prior to undertaking longer-term interventions, is one way to address this. Thus, whilst this work does not explicitly relate to investigating social processing in atypical groups, it is a necessary precursor.

#### Stimulus properties

Several studies have used NIBS methods to investigate social processing in autism (e.g. Théoret *et al.*, 2005; Enticott *et al.*, 2012). For example, Théoret *et al.* (2005) demonstrated a reduced MEP response to observed actions in participants with autism relative to neurotypical controls. One explanation for these results may be that participants with autism show a reduced mirror response to observed actions. However, this reduced response may also be due to the type of stimuli used. Specifically, if stimuli presented do not adequately map onto motor representations in the brains of participants with autism, this may also present as a reduced MEP response. One reason for this may be that participants with autism move differently to neurotypical controls. For example, participants with autism have a different kinematic profile when executing intransitive movements compared to neurotypical controls (Cook *et al.*, 2013). Considering such differences when designing stimuli is important to allow stronger inferences to be drawn. In the case of action observation, this could simply involve inclusion of movements made by autistic and non-autistic individuals, as well as several movements made by the participant themselves.

### Understanding NIBS–medication interactions

Many cognitive studies using NIBS typically exclude participants taking psychotropic medications based on safety criteria from Rossi *et al.* (2009). In neurotypical adults, this is important to reduce noise in the data and to ensure participant safety. However, this approach is less straightforward in atypical groups. It is common to decide on exclusion based on contraindications to NIBS by assessing the cost/benefit ratio of participant involvement in the study. Whilst this may be a good approach for therapeutic interventions targeting treatment-resistant disorders, it does limit inclusion of participants in research investigating atypical groups. Approximately 60% of participants with autism are taking one or more psychotropic medications (Buck *et al.*, 2014). Therefore, we need to understand safety and efficacy of NIBS in combination with these drugs to prevent sampling bias when testing atypical groups. Due to high heterogeneity, ensuring that study findings reflect the wider cohort is essential in order to interpret cognitive processes in atypical groups.

This is particularly important when investigating social processing, as people may be on medication to ameliorate social deficits. Excluding such participants from studies investigating social processing can therefore bias the sample tested. McLaren *et al.* (2018) reviewed the interaction between medications and tDCS effects over M1 in neurotypical adults. Among others, interactions between drugs that alter neurotransmitter concentrations (e.g. GABA and dopamine) and the effects of tDCS were observed. The authors highlight the use of such drugs in treating neuropsychiatric conditions (e.g. anxiety and schizophrenia), and, therefore, the importance of considering such interactions when translating tDCS protocols to atypical cohorts. However, the authors also stress caution when applying such findings to an atypical cohort, given differences in brain structure and function relative to neurotypical controls as well as potential differences in response to a given drug. Support for this cautious approach comes from work by Ajram *et al.* (2017), who showed that participants with autism showed a different neural response to a GABA- and glutamate-acting drug compared to neurotypical controls. Thus, it is important to consider the way in which a drug works in an atypical group, as well as potential (differential) NIBS–medication interactions. This will be a challenging line of research requiring data beyond that collected in neurotypical controls, and such research is currently in its infancy.

One way to inform design of such studies is to use existing data from atypical groups taking part in clinical trials using NIBS. An increasing number of studies are being conducted using NIBS in participants with psychiatric disorders either as a treatment for core symptoms or to treat co-occurring disorders (e.g. for treatment of depression in participants with schizophrenia or autism). Many of these participants are also on psychotropic medications, and so, whilst not the primary aim of the research, some of these studies also include analyses looking at NIBS–drug interactions (e.g. Hoffman *et al.*, 2000). Using findings from this literature, and literature assessing NIBS–drug interactions in neurotypical participants (e.g. Rumi *et al.*, 2005; Herwig *et al.*, 2007; Liu *et al.*, 2014; McLaren *et al.*, 2018), may help us to begin to identify common interactions and safety limits of NIBS use in an atypical brain. Once these are better understood, we can then use these findings to inform the design of studies investigating other areas of cognition such as social processing.

## Conclusions

It is clear that NIBS methods have improved understanding of social processing. However, many challenges still face research into social processing in typical and atypical groups. Promising techniques (e.g. targeting deeper structures using tFUS) are emerging, and it may be possible to exploit existing knowledge of NIBS techniques (e.g. state-dependent effects of TMS) to refine methodology. Research into NIBS in typical groups can also be used to inform NIBS protocol in atypical groups when combined with advances in understanding of brain stimulation effects in different cohorts. Along with growing understanding of NIBS mechanisms in typical and atypical cohorts, advances in our understanding of social processing have brought behavioural paradigms in the field to a stage where they are accessible both conceptually and anatomically to NIBS research.

## Funding

This work was supported by a doctoral studentship from the Medical Research Council (MR/M50175X/1 to T.P.), the Leverhulme Trust (grant number PLP-2015-019 to C.C.), the Economic and Social Research Council (ES/R007527/1 to G.B. and M.J.B.) and the Baily Thomas Charitable Trust (3751-6536 to G.B.).

*Conflict of interest*: None declared.
